# Manifestations of pilocytic astrocytoma: a pictorial review

**DOI:** 10.1007/s13244-014-0328-2

**Published:** 2014-05-02

**Authors:** Danai Chourmouzi, Elissabet Papadopoulou, Manolis Konstantinidis, Vasileios Syrris, Kostas Kouskouras, Afroditi Haritanti, George Karkavelas, Antonios Drevelegas

**Affiliations:** 1 Department of Radiology, Interbalcan Medical Centre, Thessaloniki, Greece; 2 Department of Radiology, Aristotle University of Thessaloniki, “AHEPA” Hospital, Thessaloniki, Greece; 3 Department of Pathology, School of Medicine, Aristotle University of Thessaloniki, Thessaloniki, Greece

**Keywords:** Pilocytic astrocytoma, Glioma, Magnetic resonance imaging

## Abstract

**Background:**

Pilocytic astrocytoma can be challenging to diagnose.

**Methods:**

Its clinical presentations can differ, directly related to its size and location, and are relatively unreliable. Similarly, imaging findings also vary with the location of the pilocytic astrocytoma.

**Results:**

This review provides an overview of the different imaging findings regarding pilocytic astrocytomas using both conventional and advanced magnetic resonance imaging sequences according to tumour location; the findings are strongly related to the tumour’s tendency to infiltrate surrounding structures, being able to carry out gross total resection, and finally the prognosis.

**Conclusions:**

Knowledge of these imaging manifestations of pilocytic astrocytoma may be helpful to arrive at an accurate diagnosis.

***Teaching Points*:**

- *To recognise the various imaging findings of pilocytic astrocytoma on both conventional and advanced magnetic resonance imaging sequences*.
- *To identify the characteristic imaging findings according to tumour location*.
- *To discuss the relevant differential diagnoses of pilocytic astrocytoma in each tumour location*.

## Introduction

Pilocytic astrocytoma (PA) is a rare, slow-growing glioma, classified as grade I by the World Health Organisation (WHO); it typically occurs in children and young adults [1, 2]. PA is the most common glial neoplasm in children. Only one-third of patients are older than 18 years of age and only 17 % are older than 30 years of age. In the paediatric population, two-thirds of lesions are located in the cerebellum; in adults, one-half of tumours are supratentorial [3, 4]. The cerebellum and the region around the third ventricle are the most common sites of origin; however, the entire neuraxis can be affected, with a preference for the optic nerve, optic chiasma, hypothalamus, cerebellum, brainstem, thalamus, basal ganglia, and cerebral hemispheres [5–7].

There are no clinical features that are unique to these tumours. Signs and symptoms are usually of several months’ duration and are directly related to the size, location, and presence of associated hydrocephalus. PAs usually follow an indolent course, with an extremely high survival rate—over 90 % at 10 years of age [8].

**Fig. 1:**
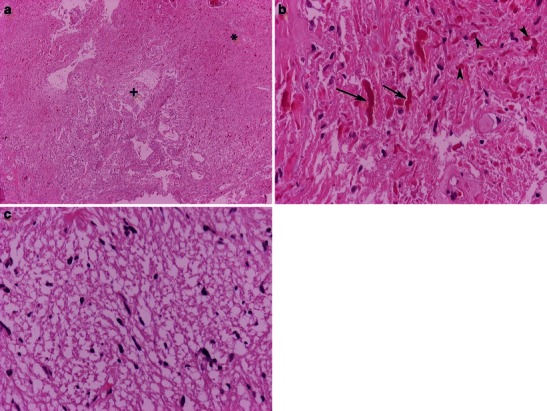
Pilocytic astrocytoma is characterised by **a** biphasic compact (*) and looser, microcystic areas (+); haematoxylin & eosin (H&E) 40×. **b** High magnification of eosinophilic granular bodies (arrowheads) and Rosenthal fibers (arrows); H&E 400×. **c** Higher magnification of the loose area; H&E 100×

The macroscopic appearance of PAs is typically well-circumscribed, cystlike masses with a discrete mural nodule [9]. The name “pilocytic” (directly translated as “hair cell”) is derived from the long, hair-like projections that emanate from the neoplastic astrocytes. Microscopically, this tumour often reveals a biphasic pattern in which more compact areas composed of bipolar areas and brightly eosinophilic Rosenthal fibers alternate with looser, spongier areas with prominent microcysts. Eosinophilic granular bodies are found in both compact and loose areas (Fig. 1a-c). Pleomorphism, infrequent mitosis, or vascular proliferation may be observed; however, unlike other astrocytomas, they are rather degenerative and do not have an ominous prognosis. Hyalinisation of the blood vessels is another feature of PAs. Microvascular proliferation of PAs accounts for the contrast enhancement that accompanies these tumours on cross-sectional imaging.

A PA with anaplastic features often contains more than four mitoses per ten high-powered fields, as well as pseudopalisading necrosis. While PAs with anaplastic features do not have defined criteria under the WHO classification, evidence suggests that PAs with anaplastic features and pseudopalisading necrosis behave similarly to WHO grade 3 neoplasms.

**Fig. 2:**
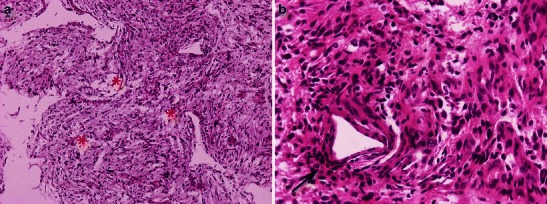
Pilomyxoid astrocytoma. **a** Piloid cells in a loose myxoid stroma (asterisks); H&E 100×. **b** Perivascular arrangement of neoplastic cells (arrow); 400×

Pilomyxoid astrocytoma (PMA) is a recently described astrocytic tumour that has been previously diagnosed as pilocytic astrocytoma. PMA can be defined as either a variant of PA or a separate entity with monomorphous pilomyxoid characteristics. PMA is a grade II tumour characterised by a prominent myxoid stroma and angiocentric arrangement of neoplastic cells (Fig. 2a, b). More aggressive than PA, PMA more often exhibits local recurrence, as well as cerebrospinal spreading [10].

When imaged in cross section, two-thirds of all PAs demonstrate a classic appearance: a cystic mass with an enhancing mural nodule. However, less common appearances are quite nonspecific. A feature specific of this benign neoplasm is that in many cases it displays histological and imaging features that are commonly seen in higher grade neoplasms and appear incongruous for a slow-growing brain tumour with fairly bland histological characteristics, such as microvascular proliferation, infiltration of surrounding tissues and structures, intratumoral haemorrhage, intense enhancement on post-contrast images, and leptomeningeal dissemination [2, 9, 11–13].

Although gross total resection of the tumour is often curative, location in critical or deep areas (such as the brainstem and hypothalamus) can render it unresectable and require additional management approaches. Strategies for treating PA in critical or deep areas, such as chiasmatic and brainstem PAs, include observation and combinations of surgery, chemotherapy, and radiotherapy. Although pathological diagnosis remains the gold standard, preoperative diagnosis of tumour behaviour and extent is of utmost importance in selecting the appropriate therapeutic management in every single case [14]. Quite often, the diagnosis of PA is initially made or suggested on the basis of an imaging study. Therefore, it is important for all radiologists to be aware of the many clinical and radiological manifestations of this tumour type.

The aim of this pictorial review is to illustrate the wide spectrum of radiologic manifestations of PAs on both conventional and advanced magnetic resonance imaging (MRI) sequences according to tumour location.

## Infratentorial manifestations

### Cerebellar PA

Cerebellar PA (CPA) is the most common cerebellar tumour in children [15]. The peak incidence of CPA is between the ages of 5 and 13 years. CPA occurs with equal frequency in boys and girls. Although CPA is usually sporadic, associations with neurofibromatosis type 1, Turcot syndrome, PHACE(S) syndrome, and Ollier’s disease have been reported [16].

There is contradictory evidence in the literature regarding the most common location of PA in the cerebellum. In one series of 132 patients, 16 % of tumours arose in the vermis, 53 % in the cerebellar hemisphere, and 26 % in both; 34 % also involved the brainstem [17]. However, in another review of 168 cases, 71 % of PA tumours were located in the vermis, while 29 % occurred in the hemisphere [18].

Because PA growth is generally slow, affected children usually present with a long history of waxing and waning signs of increased intracranial pressure. Headache, vomiting, gait disturbance, blurred vision, diplopia, and neck pain are common symptoms in patients with CPA [16].

**Fig. 3:**
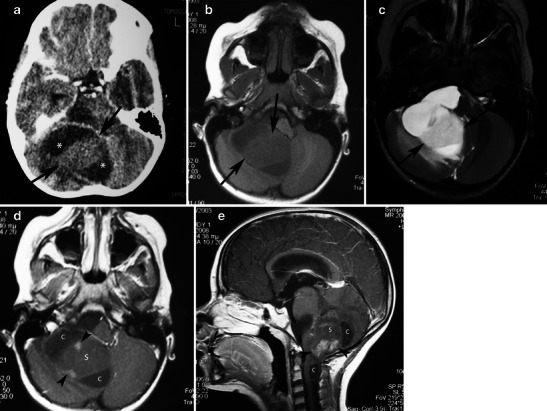
Juvenile pilocytic astrocytoma of the cerebellum in a 5-year-old girl. **a** Post-contrast CT shows a cystic lesion involving the right cerebellar hemisphere with a hypodense cystic component (asterisks) and a large, solid, isodense component (arrows). **b** On axial T1-weighted image, the solid component is homogeneous and hypointense compared with the grey matter (arrows). **c** On T2-weighted images, the solid component appears hyperintense compared with the grey matter and slightly hypointense compared with the cerebrospinal fluid (arrows). Post-contrast axial (**d**) and sagittal (**e**) images demonstrate heterogeneous enhancement of the solid component of the mass with areas that remain unenhanced (s) and areas with nodular enhancement (arrowheads). Note the cystic components (**c**)

**Fig. 4:**
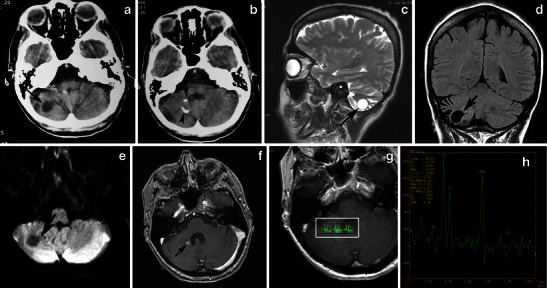
A pilocytic astrocytoma of the right cerebellar hemisphere incidentally discovered during staging in a 45-year-old woman with breast cancer. **a** An axial CT image shows a well-marginated cyst in the right cerebellar hemisphere (arrow). **b** An axial CT image in a higher level shows fleck-like calcifications (arrowheads). On sagittal T2-weighted (**c**), coronal FLAIR (**d**), and axial diffusion-weighted (**e**) images, the cyst follows cerebrospinal fluid intensity (arrows). This axial post-contrast T1-weighted image (**f**) depicts an enhancing focus (arrow). On MRS centred on an enhancing nodule with TE 144-ms (**g**, **h**), there is mild elevation of choline/Cr, mild reduction of NAA/Cr, and a small lactate peak

CPAs can be small or very large, and cystic, solid, or solid with a necrotic center. Typical computed tomography (CT) imaging features are a large and predominantly cystic mass arising from the vermis cerebelli or the cerebellar hemispheres. The solid part appears hypodense or isodense (or, less commonly, hyperdense) and enhanced on post-contrast images [18, 19] (Fig. 3a). Tumour calcification is rare, but tends to resemble flecks when it does occur [15] (Fig. 4a,b).

**Fig. 5:**
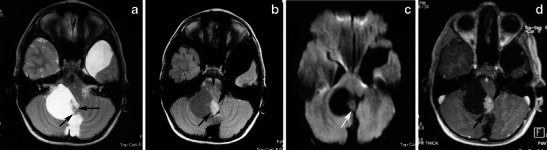
A large, cystic CPA in a 7-year-old boy. **(a)** An axial T2-weighted image shows a hyperintense mass of the right cerebellar hemisphere with a less intense soft-tissue nodule along its medial margin and without surrounding oedema (arrows). Note the arachnoid cyst in the left middle cranial fossa. **b** On an axial FLAIR image, the cystic component shows low signal intensity that is higher than that of the CSF, and the soft tissue nodule is homogeneous and slightly hyperintense (arrows). **c** On a diffusion-weighted image, the mural nodule appears isointense (arrow). **d** An axial, contrast-enhanced, T1-weighted image demonstrates intense enhancement of the mural nodule (arrows)

**Fig. 6:**
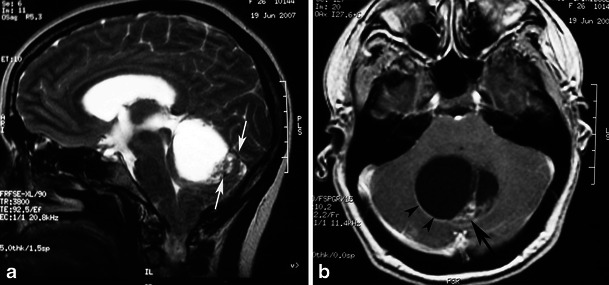
Pilocytic astrocytoma in a 26-year-old woman arising in the vermis. **a** A sagittal T2-weighted image shows a hyperintense cystic mass of the cerebellar vermis with a peripherally located mural nodule (arrows) compressing the fourth ventricle. **b** An axial contrast-enhanced T1-weighted image demonstrates intense enhancement of the mural nodule (arrow), as well as cyst wall enhancement (arrowheads)

**Fig. 7:**
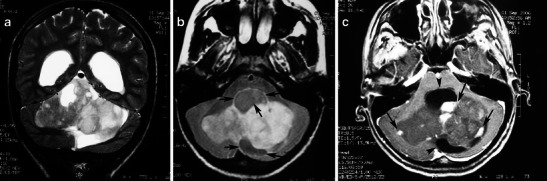
A pilocytic astrocytoma with a predominantly solid mass and a minimal cyst-like component in a 14-year-old girl. **a** This coronal T2-weighted image shows a large, well-marginated mass, involving the vermis and both cerebellar hemispheres that effaces the fourth ventricle. **b** On an axial FLAIR image, the solid mass shows high signal intensity with hypointense cystic areas (arrows). **c** On an axial contrast-enhanced image, the mass shows inhomogeneous contrast enhancement, with nodules that exhibit intense enhancement (arrows) and other cystic areas that remain unenhanced (arrowheads)

**Fig. 8:**
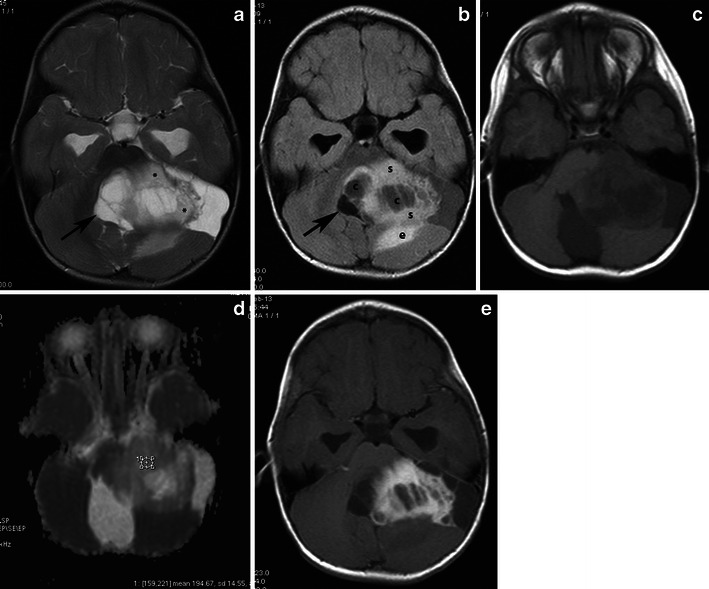
Pilocytic astrocytoma of the left cerebellar hemisphere with a solid and cystic component severely compressing the fourth ventricle and the medulla, producing obstructive hydrocephalus. **a** An axial T2-weighted image shows a cystic, hyperintense mass with a less intense solid component (asterisks) compressing the fourth ventricle (arrow). **b** On a FLAIR image, the cystic component (**c**) shows low signal intensity, which is higher than that of the CSF, and the solid component (s) shows high signal intensity. Note the low signal of the compressed fourth ventricle (arrow) and the peritumoral oedema (**e**). **c** On this T1-weighted image, the solid components appear hypointense compared with grey matter. **d** The solid component shows high values on an ADC map. **e** Intense enhancement of solid components is depicted after the administration of a paramagnetic agent

The appearance of PA on MRI is variable and depends on the tumour’s size and structure. Four predominant imaging patterns of PA have been described: a mass with a non-enhancing cyst and an intensely enhancing mural nodule (Figs. 4 and 5); a mass with an enhancing cyst wall and an intensely enhancing mural nodule (Fig. 6); a necrotic mass with a central non-enhancing zone; and a predominantly solid mass with minimal to no cystlike component [9] (Figs. 7 and 8). Solid, non-necrotic tumours are less common and account for approximately 10 % of cases. However approximately 50 % of the tumours are cystic, with a mural nodule attached to one part of the cyst wall [15] (Fig. 5).

The solid component of PAs can be homogeneous or heterogeneous. It is usually hypo- to isointense on T1-weighted MRI and hyperintense on T2-weighted/FLAIR images compared with grey matter. Compared with the cerebrospinal fluid, the solid component is isointense on T2-weighted images and hyperintense on FLAIR images [20]. After the administration of a paramagnetic agent, the solid component of PAs exhibits variable enhancement patterns: areas with prominent enhancement as well as areas that remain unenhanced. Yet the cystic component appears isointense on T1- and T2-weighted images and hyperintense on FLAIR images compared with cerebrospinal fluid (CSF) signal intensity. Enhancement of the cyst wall is not always present and is not necessarily indicative of tumour involvement. Haemorrhages have rarely been reported in PAs and are usually not massive.

On diffusion-weighted imaging, cystic components of the mass exhibit the diffusion properties of CSF. The solid component displays high apparent diffusion coefficient (ADC) values, which indicate the low cellularity of PA [21]. Pavlisa et al. report that ADC values greater than 120 × 10^−5^ mm^2^/s are indicative of PA, while an ADC between 80 and 120 × 10^−5^ mm^2^/s is characteristic of ependymoma. Myeloblastoma usually exhibits strong restriction, with ADC less than 80 × 10^−5^ mm^2^/s [22] (Figs. 4e, 5c and 8d).

Reported studies that employ magnetic resonance spectroscopy (MRS) to examine CPA have revealed high choline, high lactate, and low N-acetylaspartate (NAA) levels, features that are usually associated with high-grade tumours. The presence of lactate within pilocytic tumours might be explained by several mechanisms, such as an abnormal number or dysfunction of mitochondria (which would interfere with the processes of oxidative phosphorylation and electron transport); alterations in proportional oxygen delivery, oxygen extraction, or oxygen usage by the tumour; or anaerobic glycolysis by tumour cells [23, 24]. However, an in vivo study of PAs using proton MRS by Sutton et al. reported a choline/NAA ratio of 1.80, which is lower than the ratio observed in other studies [25] (Figs. 4g,h). Although investigators report that cerebellar PA with anaplastic features is more often solid than cystic from a neuroimaging standpoint, CT and MRI findings are inadequate to predict these histologic features.

Differential diagnosis of CPA in children mainly includes ependymoma and medulloblastoma. Ependymomas might have calcifications (50 %) and tend to extend laterally to the cerebellopontine angles, while medulloblastomas tend to disseminate through the CSF spaces to the brain and spine. Rumboldt et al. [21] reported the contribution of ADC in the differentiation of paediatric cerebellar tumours and proposed that ADC values >1.4 are more consistent with PA, while values <0.9 are more suggestive of medulloblastoma. In adults, the differential diagnosis of a cerebellar partially cystic mass should also include metastasis (usually known primary) and haemangioblastoma, apart from PA.

Investigators have demonstrated marked elevation of relative cerebral blood volume (rCBV) in cases of haemangioblastoma and mild elevation in cases of PA, with statistically significant differences. Kumar et al. reported mean relative cerebral blood volumes of 7.7 in patients with haemangioblastomas and 1.8 in patients with PAs. Therefore, a finding of elevated rCBV levels should lead to a high preoperative suspicion of haemangioblastoma [26].

Gross total resection is the treatment of choice for CPA and is often curative. Progression of residual disease can be controlled by tumour re-excision and stereotactic radiosurgery [27, 28]. In the event of biopsy-proven pilocytic astrocytoma with anaplastic features, radiation therapy and/or chemotherapy will be required [12].

### Brainstem PA

Brainstem tumours (BSTs) account for 10–15 % of primary intracranial tumours in children. They occur at a mean age of 7 years and are usually divided between diffuse BSTs and non-diffuse or focal BSTs. BSTs are almost exclusively astrocytomas. Diffuse BSTs are usually fibrillary astrocytomas or malignant neoplasms, while focal BSTs are mainly PAs.

PA of the brainstem (BPA) in children can be located in the midbrain (in the pons), but is more frequently located in the medulla. Involvement of the upper cervical cord is sometimes noted [29–31]. Adult BPA is very rare. Guillamo et al. [32] report only a single case of PA in 32 biopsied adult brainstem gliomas.

BPA expands the brainstem and is typically associated with an exophytic growth, usually from its dorsal surface [20, 31]. It is characterised by sharp margins with cyst-like areas. The dorsal extension is a result of the inability of the low-grade tumour to infiltrate caudally below the decussations of the corticospinal tracts and medial lemnisci at the craniocervical junction. Therefore, dorsally exophytic tumours should be considered focal BSTs. However, ventral exophytic PAs have also been observed. Diffusely infiltrative brainstem gliomas are usually fibrillary astrocytomas located in the ventral pons, which present with abducens palsy, often engulf the basilar artery, and are associated with a grim prognosis [32]. Symptoms and signs are usually related to increased intracranial pressure. Nausea, vomiting, and ataxia with evidence of torticollis, papilledema, and nystagmus palsies of the sixth and seventh cranial nerves are usually observed at physical examination.

**Fig. 9:**
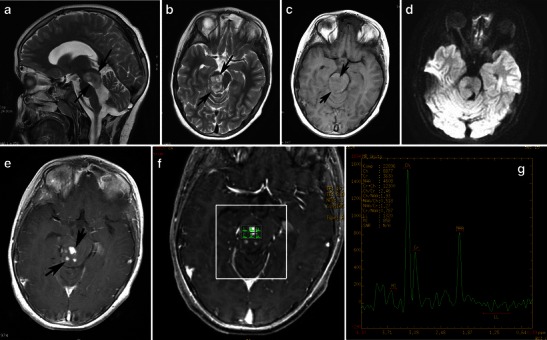
Focal midbrain glioma, a histopathologically proven pilocytic astrocytoma in an 11-year-old boy. Sagittal (**a**) and axial (**b**) T2-weighted images show an exophytic, sharply marginated, hyperintense mass involving the right cerebral peduncle. No surrounding oedema is identified. On this T1-weighted image (**c**), the mass is isointense compared with grey matter. The mass is isointense on diffusion-weighted imaging (**d**). An axial post-contrast image (**e**) shows heterogeneous enhancement of the tumour. Proton MRS with TE 144-ms (**f**, **g**) revealed high choline/Cr (2.46) and NAA/Cr ratios (1.27)

**Fig. 10:**
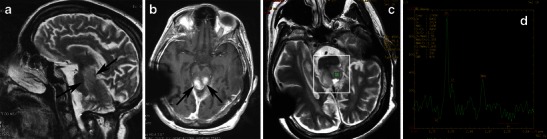
Midbrain pilocytic astrocytoma in a 20-year-old woman. A sagittal T2-weighted image (**a**) shows exophytic growth of the tumour. An axial, post-contrast, T1-weighted (**b**) image shows intense contrast enhancement (arrows). On proton MRS with TE 144 ms (**c**, **d**), "pseudomalignant" spectrum is observed with an elevated choline peak and reduction of the NAA peak, choline/Cr ratio (3.17), and NAA/Cr ratio (2.55)

**Fig. 11:**
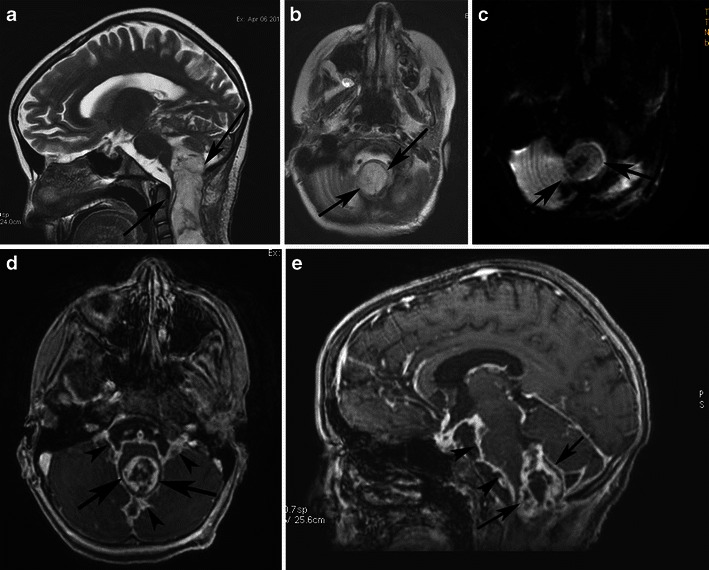
A pilocytic astrocytoma extending to the upper cervical spine with dissemination after partial resection. **a** Sagittal T2-weighted and **b** axial T2-weighted images at the level of the foramen magnum show a high-signal cyst-like mass (arrows). **c** The lesion is hypointense on diffusion-weighted imaging (arrows). Contrast-enhanced axial (**d**) and sagittal T1-weighted (**e**) images of the brain show inhomogeneous enhancement of the mass (arrows), as well as diffuse enhancement of the basilar cisternal spaces (arrowheads)

On CT BPA presents as a heterogeneous mass that expands the pons, distorts the fourth ventricle, and results in a degree of hydrocephalus. The mass is predominantly of low attenuation, with areas of intense enhancement on postcontrast images. BPAs are very bright on T2-weighted MRI. Thus, they can be differentiated from ependymomas, which are isodense to grey matter on CT and isointense on T2-weighted MRI. In addition, dorsally exophytic tumours have been observed to push the fourth ventricle upward, in contrast to ependymomas, which originate within the wall of the fourth ventricle and expand it. After the administration of contrast media, BPA exhibits inconsistently homogeneous or heterogeneous enhancement (Figs. 9, 10, and 11).

Proton MRS and perfusion imaging may be useful to differentiate low-grade PA from high-grade tumours in that lower grade glial tumours have lower choline peaks and smaller blood volume than higher grade glial tumours (Fig. 9f,g). However, pilocytic astrocytomas may have relatively high choline and lactate peaks and relatively high blood volumes, thus mimicking high-grade tumours (Fig. 10c,d). The distinction between the more biologically aggressive fibrillary astrocytoma and PA is more than an academic curiosity, since the fibrillary brainstem astrocytoma is associated with a dismal prognosis compared with the excellent outlook associated with the pilocytic variety [33].

Management of BPA is a difficult challenge. Surgical treatment must cope with the difficulties of both deep location and eloquent area tumours. The tumour can be controlled by partial surgical removal, and a residual tumour can sometimes decrease in size after surgery. Gross total removal of these tumours, although difficult, may be performed [34].

BPA is associated with a relatively good prognosis in children and adults [32, 35]. Ahmed et al. recently reported long-term overall survival of 14.8 years in a series of 48 children, with respective 1-, 5-, and 10-year overall survival rates of 85 %, 67 %, and 59 % [36].

## Supratentorial manifestations

### Chiasmatic/hypothalamic PA

Gliomas originating in the optic chiasm often enlarge and involve the hypothalamus; similarly, astrocytomas arising in the hypothalamus often grow anteriorly and inferiorly to involve the optic chiasm. Because the primary site of origin cannot be determined in many cases, tumours arising from these two locations are often discussed together. Astrocytomas of the optic chiasm and hypothalamus make up 10 % to 15 % of supratentorial tumours in children; males and females are approximately equally affected. One-third of patients have neurofibromatosis type 1 (NF1) [37].

The most common symptom of hypothalamic/optic pathway PA is diminished visual acuity. Endocrine dysfunction, most commonly short stature secondary to reduced growth hormone, is usually observed. Large tumours produce hydrocephalus secondary to extension into the anterior third ventricle and obstruction at the level of the foramen of Monro.

**Fig. 12:**
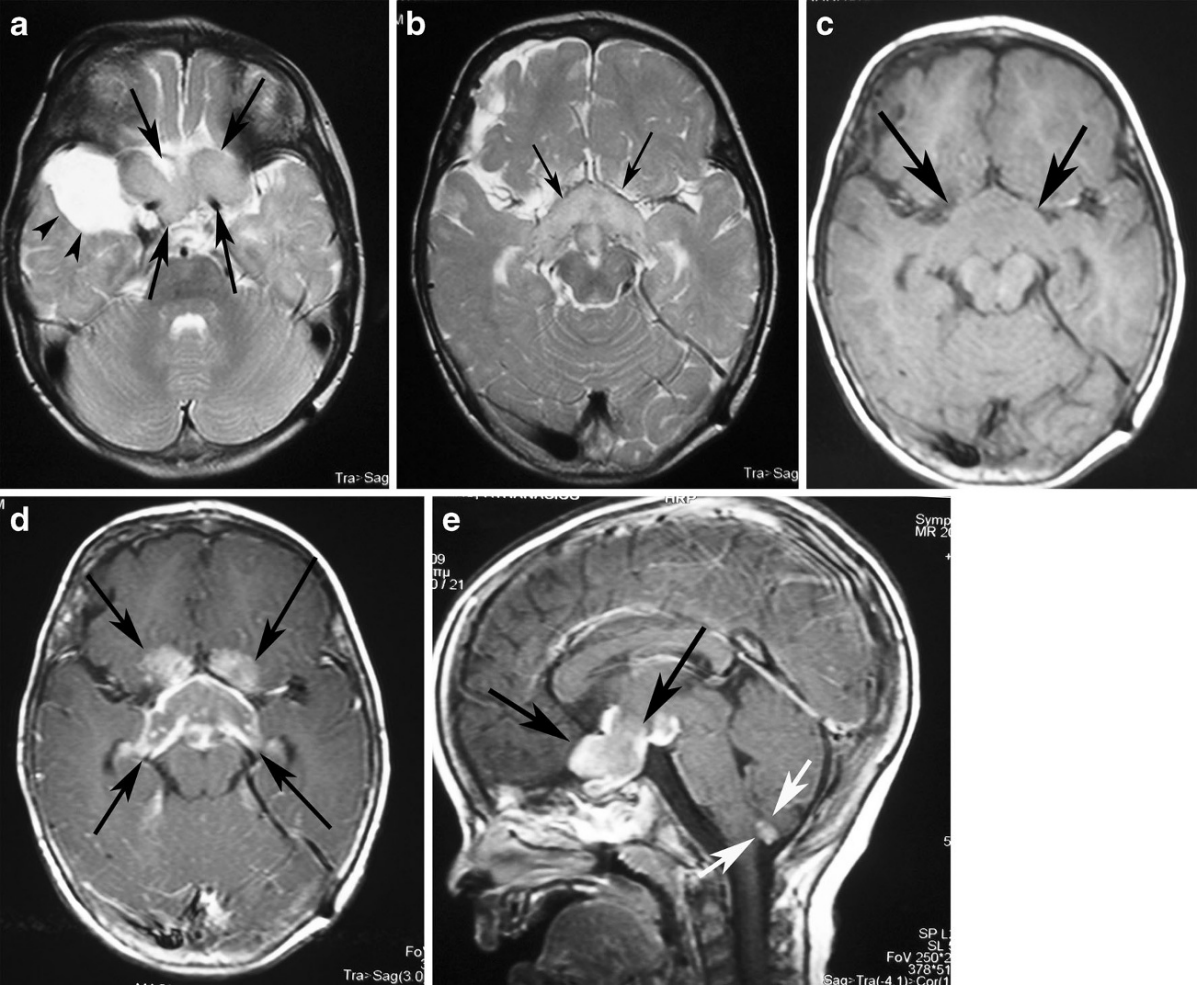
A pilocytic astrocytoma involving optic pathways. **a** An axial T2-weighted image shows a large mass originating in the region of the optic chiasm/hypothalamus (arrows). Note the arachnoid cyst in the right middle cranial fossa (arrowheads). **b** An axial T2-weighted image in a higher level shows the hyperintense tumour extending to the optic radiations (arrows). On this T1-weighted image (**c**), the mass is isointense compared with grey matter. T1-weighted axial (**d**) and sagittal (**e**) post-contrast images show heterogeneous enhancement extending anteriorly towards the optic nerves and posteriorly towards the optic tracts. Note the enhancing nodule dorsal to the medulla due to dissemination of the pilocytic astrocytoma (arrows)

MRI provides precise demarcation of tumour extension and in many cases allows accurate preoperative diagnosis based on MRI findings [38]. Involvement of the optic pathway causes enlargement of the optic chiasm, nerve, and tracts. Chiasmatic/hypothalamic PAs are hypointense on T1-weighted sequence and hyperintense on T2-weighted and FLAIR sequences. When large and bulky, tumours are typically heterogeneous and mainly solid, with mild or marked enhancement after the intravenous administration of contrast media (Fig. 12). Using fat-suppression techniques to eliminate fat signal from the skull base can increase the value of contrast-enhanced MR images of this area.

Cystic areas and trapped pools of CSF are sometimes noted. The indolent growth rate of chiasmatic/hypothalamic PA and its tendency to infiltrate along the arachnoid with secondary fibrotic changes might explain the secondary development of arachnoid cysts (Fig. 12). This phenomenon has not been reported in association with other brain tumours [9].

Differential diagnosis in children should include craniopharyngioma, germinoma, and Langerhans cell histiocytosis. In adults, differential diagnosis should include pituitary macroadenoma, sellar meningioma, craniopharingioma, metastases (more often from breast and lung primary tumours), and lymphoma [39].

**Fig. 13:**
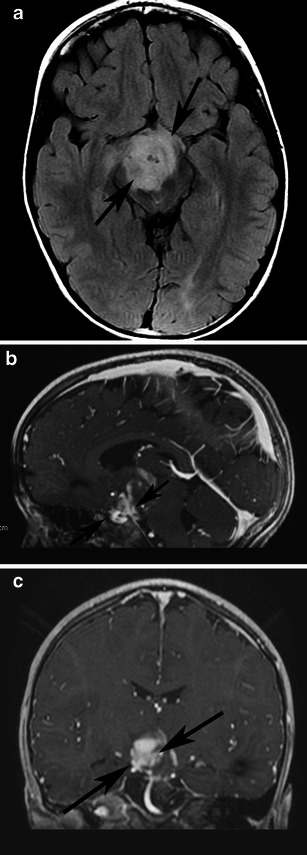
Pilomyxoid astrocytoma in a 5-year-old girl. **a** An axial FLAIR image shows a large solid lobulated suprasellar mass with uniform hyperintensity (arrows). Postgadolinium sagittal (**b**) and coronal (**c**) T1-weighted images reveal a homogeneously enhancing suprasellar infiltrative tumour (arrows)

PMA is a rare variant of PA. It develops more frequently in the suprasellar area. PMAs are more aggressive masses and are mainly solid with uniform or heterogeneous rim enhancement. They exhibit higher rates of intratumoral haemorrhage and frequent local recurrence, and are often associated with leptomeningeal dissemination [40] (Fig. 13). Chiasmatic/hypothalamic PMAs reportedly display higher choline/Cr ratios outside their enhancing tumour margins, which likely reflects their more aggressive behaviour [41].

The goal of surgery in cases of chiasmatic/hypothalamic PA is primarily to establish a histological diagnosis; if a typical low-grade astrocytoma is encountered, then limited resection is performed. Large masses that obstruct the foramen of Monro are debulked to relieve ventricular obstruction. The majority of such patients respond to platinum-based chemotherapy. Chemotherapy can also improve serious visual dysfunction, and a chemotherapy-first strategy can preserve the cognitive outcome of patients, who thereby avoid the need for radiotherapy [42]. Chemotherapy and radiation therapy can provide good control of the disease, with 5-year overall survival reported at >90 % [43]. Even when leptomeningeal gliomatosis presents, craniospinal irradiation and temozolomide can be very effective in disease control [44].

### Optic nerve PA

Anterior optic pathway gliomas are usually PAs presenting during the first decade of life. More than one-half of patients have NF1 [45]. Segal et al. [46] reviewed 331 patients with NF1 and reported the development of optic nerve glioma in 13 % of them. Bilateral tumours are almost pathognomonic for NF1.

Anterior optic pathway PA exhibits different biological behaviour in patients with and without NF1. Isolated optic involvement, preservation of the optic nerve configuration, smaller tumour size, absence of symptoms, stability or slow growth, and even spontaneous regression are more often observed in patients with NF1. In comparison, chiasmal involvement, altered optic nerve configuration, greater tumour size, presence of symptoms, and local growth are more often encountered in patients without NF1 [45, 47].

**Fig. 14:**
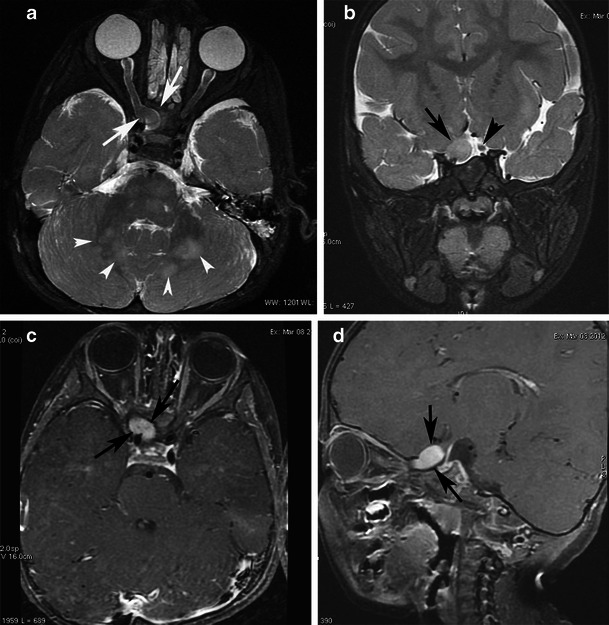
A pilocytic astrocytoma of the optic nerve in a 3-year-old girl with NF1. **a** An axial T2-weighted image reveals characteristic kinking of the enlarged right optic nerve within the intraconal compartment and enlargement (arrow) of the posterior extraconal compartment. Note the characteristic NF1 bright lesions in the cerebellar hemispheres (arrowheads). **b** A coronal STIR image shows the enlarged right optic nerve (arrow) in comparison with the normal left optic nerve (arrowhead). On these contrast-enhanced axial (**c**) and sagittal (**d**) T1-weighted images, intense enhancement of the extraconal component is observed (arrows)

MRI displays fusiform enlargement of the optic nerve, usually with kinking or tortuosity and contrast enhancement after the intravenous administration of gadolinium (Fig. 14). Infiltration of the perineural subarachnoid space, cystic components, and optic canal enlargement are sometimes observed.

Treatment is required only in symptomatic, growing tumours. Resection is reserved for eyes with poor or absent vision, for cosmetic reasons, or because of exposure keratopathy. Radiotherapy and chemotherapy can be used to control tumour growth; however, radiotherapy should be avoided in patients younger than 5 years of age [48].

The prognosis for anterior optic pathway gliomas is very good; a 10-year progression-free survival rate of 72 % has been reported (in contrast to 58 % for posterior optic pathway gliomas) [49].

### Hemispheric PA

Among the various age groups of patients with PA, the major tumour location differs in prevalence. In children, up to 67 % of patients present with cerebellar lesions; in adults, approximately 55 % of patients present with supratentorial lesions. Although juvenile PAs are less common in the cerebral hemisphere, they constitute 20 % of supratentorial brain tumours.

Headache, seizure activity, hemiparesis, ataxia, nausea, and vomiting are common clinical manifestations of hemispheric pilocytic astrocytomas (HPAs). The occurrence of seizure activity generally indicates cortical grey matter involvement [9]. The tumours have no characteristic location, although in the paediatric population they tend to occur deep within the hemispheres and involve the basal ganglia or thalamus more frequently than do adult tumours.

**Fig. 15:**
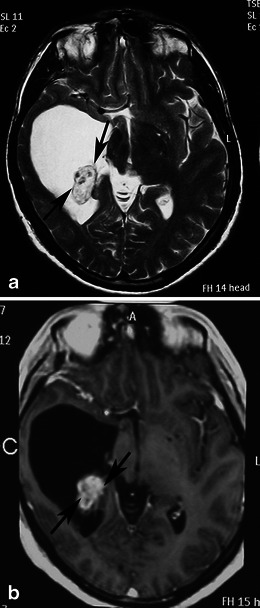
Classical appearance of a hemispheric pilocytic astrocytoma in a 45-year-old man. **a** An axial T2-weighted image demonstrates a hyperintense cystic component and a less hyperintense solid nodule within the lateral ventricle (arrows). **b** A contrast-enhanced axial T1-weighted image reveals intense enhancement of the solid nodule (arrows) and lack of enhancement of the cystic portion

**Fig. 16:**
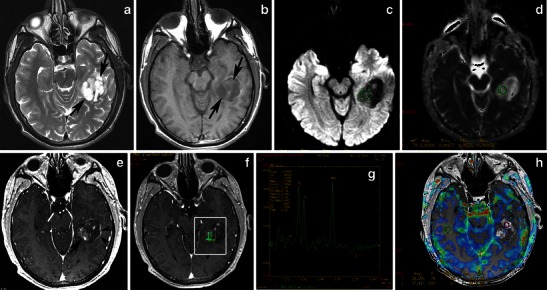
Hemispheric pilocytic astrocytomas in a 26-year-old man. **a** An axial T2-weighted image shows a temporally located, lobular mass that is very hyperintense (arrows) without vasogenic oedema. **b** On this T1-weighted image, the lesion is hypointense compared with grey matter (arrows). **c** The lesion exhibits low signal on diffusion-weighted imaging, with high signal intensity on an ADC map (**d**). **e** On this post-contrast, T1-weighted image, nodular (arrow) and peripheral rim enhancements (arrowheads) are visible. **f**, **g** MRS with TE 144 ms revealed an elevated choline peak without reduction of the NAA peak. **h** Perfusion MRI reveals low rCBV in the solid (enhanced) part of the tumour

**Fig. 17:**
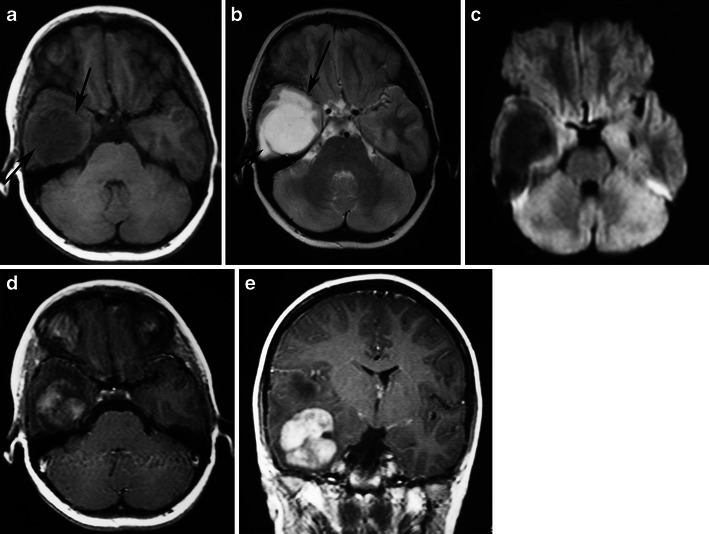
Supratentorial pilocytic astrocytoma with solid enhancement and vasogenic oedema. **a** An axial T1-weighted image shows a well-circumscribed, hypointense cerebral mass (arrows). **b** An axial T2-weighted image shows a hyperintense mass. Note the vasogenic oedema around the mass (arrows). **c** The lesion exhibits low signal on diffusion-weighted imaging. Contrast-enhanced axial (**d**) and (**e**) T1-weighted images demonstrate inhomogeneous enhancement of the mass

The most characteristic imaging finding is a cystic mass with an enhancing mural nodule, usually contiguous with the ventricular system. In one-half of HPA cases, the nodule has a deep location (Fig. 15). However, HPAs usually appear as medially located masses that are very hyperintense on T2-weighted and FLAIR images. They elicit little or no vasogenic oedema in the surrounding white matter and enhance after the injection of gadolinium [49]. The enhancement of the solid component of PA may be due to rich vascularity or to focal disturbance of the blood–brain barrier [50] (Figs. 16 and 17). Spontaneous intratumoral haemorrhage has also been described [51]. Mixed pilocytic and fibrillary astrocytomas are encountered; malignant transformation of a PA rarely occurs [6]. On diffusion-weighted imaging, solid areas of HPA have higher ADC values than high-grade hemispheric tumours [52] (Figs. 16c and 17c).

Previous reports of MRS performed on solid portions of PA tumours have documented elevations in the choline-to-NAA ratio [53] to a lesser extent than in higher grade neoplasms (Fig. 16f,g). The well-known positive correlation between increased choline levels and the glioma grade appears to be violated by WHO grade I PAs, which exhibit a wide range of choline values, with a marked increase in some cases. No significant differences have been identified in MRS metabolite profiles between paediatric and adult PAs [54].

PAs have a low maximum rCBV ratio (<1.5) and exhibit a characteristic type of signal intensity time curve with an increase in signal intensity above baseline caused by the massive leakage of contrast medium into the interstitial space [51] (Fig. 16h). Differential diagnosis should include other glial tumours, as well as metastases in adults and primitive neuroectodermal tumours in children. While high-grade gliomas are usually characterised by irregular margins and perifocal oedema, haemorrhagic features have also been encountered. Fibrillary astrocytomas, oligodendrogliomas, and gangliogliomas usually do not display the marked enhancement observed in PA. Calcifications have been detected in oligodendrogliomas and gangliogliomas, as well as scalloping/erosion of the inner table of the calvaria in cases of peripherally located tumours.

The first-line therapy for HPA is complete surgical resection. Adjuvant chemotherapy and radiotherapy are reserved for recurrent or progressive inoperable disease.

## Conclusion

In summary, this pictorial review presents imaging appearances and the differential diagnosis of PA according to tumour location. MRI with conventional sequences and advanced techniques provides useful information regarding characteristics of this low-grade tumour. High ADC values can be used in the clinical setting as a reliable factor indicating low cellularity of PA. Metabolite ratios of MRS and rCBV measurements on perfusion exhibit inferior diagnostic performance, since pilocytic astrocytomas may have relatively high choline and lactate peaks and relatively high blood volumes, thus mimicking high-grade tumours. Recognition of radiologic features and familiarity with the clinical course and sites of potential involvement are essential for making the correct diagnosis and helpful for management decisions.
